# Reliability of MEMS in Shock Environments: 2000–2020

**DOI:** 10.3390/mi12111275

**Published:** 2021-10-20

**Authors:** Tianfang Peng, Zheng You

**Affiliations:** Department of Precision Instrument, Tsinghua University, Beijing 100084, China; ptf15@mails.tsinghua.edu.cn

**Keywords:** MEMS, shock, reliability, microstructure mechanics

## Abstract

The reliability of MEMS in shock environments is a complex area which involves structural dynamics, fracture mechanics, and system reliability theory etc. With growth in the use of MEMS in automotive, IoT, aerospace and other harsh environments, there is a need for an in-depth understanding of the reliability of MEMS in shock environments. Despite the contributions of many articles that have overviewed the reliability of MEMS panoramically, a review paper that specifically focuses on the reliability research of MEMS in shock environments is, to date, absent. This paper reviews studies which examine the reliability of MEMS in shock environments from 2000 to 2020 in six sub-areas, which are: (i) response model of microstructure, (ii) shock experimental progresses, (iii) shock resistant microstructures, (iv) reliability quantification models of microstructure, (v) electronics-system-level reliability, and (vi) the coupling phenomenon of shock with other factors. This paper fills the gap around overviews of MEMS reliability in shock environments. Through the framework of these six sub-areas, we propose some directions potentially worthy of attention for future research.

## 1. Introduction

Shock is one of the most common challenges that MEMS needs to deal with in harsh environments [1]. Among different applications, the range of the amplitude and frequency of shock loads can be very large, where the amplitude range is usually 10^1^–10^4^ g, and the frequency range is usually 10 Hz–50 kHz. Some typical shock environments are shown in Table 1. In automotive, aerospace, military and other applications, MEMS devices, as a type of semiconductor device containing movable microstructures, is subjected to failures, such as fractures and adhesions [2].

Since 2000, modeling, analysis and design of MEMS devices for shock environments have been one of the most important areas of MEMS research in relation to harsh environments. Some academic monographs have been introduced that briefly covered this topic [3,4,5]. However, there are still some key questions which have not been satisfactorily addressed, such as failure threshold prediction and quantitative calculation of reliability. The inherent reason for this challenge is that the response, failure, and reliability of MEMS microstructures under impact loads are very complex problems that involve the intersection of structural dynamics, fracture mechanics, system reliability theory, and other fields [6,7,8,9,10]. Additionally, topics, such as the material properties at the micrometer/nanometer scale, the dynamic response of the microstructure, and the impact damage of the structure, are themselves the frontier research subjects in their respective fields. These all make the in-depth study of MEMS reliability in shock environments extremely challenging.

The influence of the shock load on various types of MEMS devices could be very different. From the perspective of reliability, MEMS devices are usually classified by their movable structures, so that the MEMS belonging to the same class share similar failure phenomena and mechanisms. Generally, MEMS devices are divided into four classes according to the presence/absence of the active structure, the presence/absence of impact between structures, and the presence/absence of friction between structures [11,12], The specific classification method is shown in Table 2. The complexity of the reliability problem of MEMS devices rises in these four classes.

In relation to these four classes of MEMS, Lasse Skogstrom et al. [12] reported a comprehensive overview in 2020. The failure mechanisms are summarized in Table 3. This study illustrated that the shock load poses a failure risk to the four classes of MEMS devices. It is also pointed out that the most important failure phenomenon caused by impact loads is MEMS structural fracture (sometimes accompanied by particle contamination), followed by structural adhesion, which will be discussed in detail in the third section of this article.

The reliability of MEMS is a rather broad concept. Since 2000, many articles have reviewed the reliability of MEMS in a comprehensive way. Van Spengen (2003) [13], Huang, Yunhan (2012) [14], and Somà, A (2020) et al. [15] have summarized experimental research focused on the failure phenomena of MEMS. Tanner et al. [16] reviewed successful commercial applications of MEMS in 2009 and looked forward to the main challenges facing MEMS reliability in the future. Tanner (2007) [17], Jacopo Iannacci (2014) [18], and Hu, Yi (2014) et al. [19] discussed the reliability issue from the perspective of the product design process for manufacturing-level optimization. In 2014, Tariq Jan et al. reviewed the reliability and fatigue problems of MEMS devices with cantilever beams in harsh environments [20]. In 2017, Rafiee et al. [21] summarized the relationship found between the nonlinear effects exhibited by MEMS and its reliability. In 2019, TMI Băjenescu [22] discussed the reliability issues caused by the manufacturing process of MEMS devices. In short, current reviews have provided a panoramic summary on the failure phenomena, mechanisms, and other noteworthy issues affecting the reliability of MEMS.

However, there are a lack of review articles focusing on how the reliability of MEMS is influenced by shock environments. The scale effect of microstructure may be a partial reason behind this negligence. That is, the micron-level scale of MEMS has already made them more shock-resistant than most traditional mechanical structures [23]. More specifically, low-amplitude (10^0^–10^1^ g) and low-frequency (<20 Hz) shock loads usually cannot cause damage to the MEMS structure [24]. Therefore, for commercial-grade applications, shock loads are usually not the main threat to a system’s reliability. This has led to the success of commercial-grade MEMS products [16,25]. However, this also results in less attention being devoted to the reliability of MEMS in shock environments thus far.

**Figure 1:**
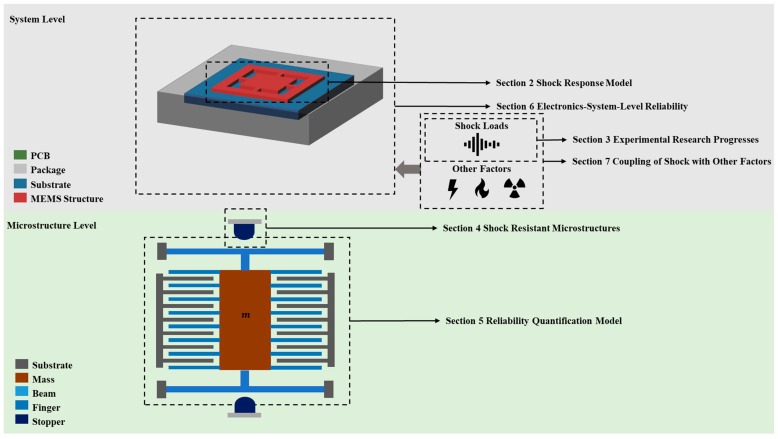
Contents of this paper.

In the recent years, applications of MEMS, such as automotive, Internet of Things, aerospace, and defense equipment applications, are gaining an increasingly large market. These applications demand much higher requirements for the reliability of MEMS devices, including severe shock environments. High-g shock loads, which feature high amplitudes (10^3^–10^4^ g) and complex frequency components (>10^2^ Hz), are the main challenge that needs to be dealt with in these applications. The response mode, failure mechanism, and reliability design of MEMS in shock environments must be considered in MEMS products in the future. Additionally, MEMS structures of highly sensitive structures, like energy harvester and bio-sensors, are vulnerable even in shock loads of low amplitude (10^1^–10^2^ g). Their reliability matters even more when being applied to healthcare equipment. Therefore, in this article, we reviewed the reliability studies of MEMS under shock loads in the past 20 years. In order to categorized these researches in a clear logic, the reliability issues covered by this paper are divided into two levels, as shown in Figure 1. The content of this paper is as follows. In the second part, we review the structural response model of MEMS under shock loads. The third part summarizes the shock experimental studies and shock excitation methods. In the fourth part, we review the anti-shock strategies in MEMS structural design. The fifth part summarizes research on the structural reliability quantification model. In the sixth part, we overview the shock experiments on the electronic-system-level. The seventh part focuses on the effect of coupling between shock loads and other physical factors.

## 2. Shock Response Model

**Figure 2:**
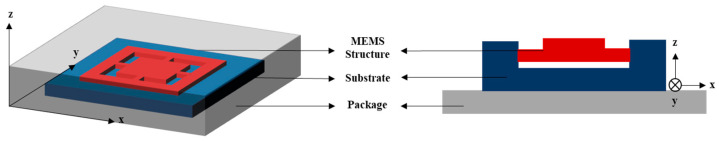
Package-substrate-microstructure model of MEMS devices.

Shock loads are often of high amplitude and short duration. Theoretically, the response of the MEMS structure under shock loads belongs to the fields of structural dynamics, vibration mechanics and shock dynamics [6,26,27]. Specifically, the acceleration pulse of the shock does not act on the entire geometric area simultaneously, but in the form of stress waves along the package-substrate-microstructure sequence, as shown in Figure 2.

Technically speaking, an accurate calculation of the response of MEMS structures under shock loads should be based on the theory of stress wave analysis, and by the transient response analysis [28,29]. However, if the stress wave analysis method is actually adopted for all kinds of shock loads and MEMS structures, it will bring huge modeling and calculation burdens. This is feasible in neither research nor product design.

Therefore, it is necessary to find a suitable theoretical model and calculation method of structural responses under shock loads. Research in this field can be classified into two categories: analytical models and numerical research.

### 2.1. Analytical Model

**Figure 3:**
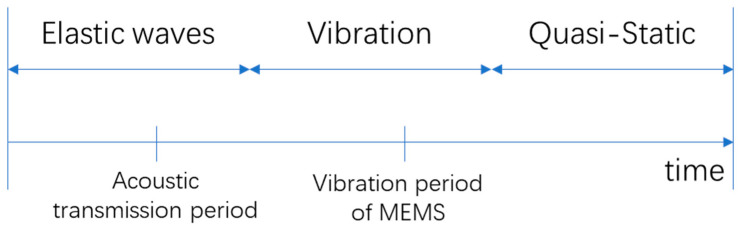
The three types of response modes [30].

In terms of the theoretical model of the response of MEMS structures to shock loads, Srikar, V. T. published groundbreaking work in 2002 [30]. Srikar proposed that the response of the MEMS structure under shock loads can be divided into three modes, as shown in Figure 3: elastic wave mode, resonance mode, and quasi-static mode, according to the relationship between shock duration, stress wave propagation time, and MEMS resonance period, respectively. Srikar proposed that, for most (nearly 90% of the cases) MEMS devices, the effect of shock loads can be regarded as quasi-static. Fang et al. further verified Srikar’s theoretical model in 2004 [31]. Many subsequent simulations on MEMS shock responses also refer to Srikar’s analytical model.

As for the shock response of the microstructure, Younis et al. have also contributed substantially. In 2005, Younis used the single-degree-of-freedom system (SDOF) with electrostatic force term to derive the analytical response of an electrostatically driven MEMS structure under half-sine, triangular and rectangular shock pulses. He indicated that the failure risk of an electrostatically driven MEMS structure under shock loads is higher, considering the pull-in effect and other potential failures [32]. In 2007, Younis et al. proposed a method that can quickly calculate the shock response of MEMS structures based on Galerkin’s reduced-order model. This paper furtherly pointed out that, for MEMS structures with low natural frequencies, calculating the impact load as a quasi-static force may lead to erroneous results. Therefore, the impact load and the amplitude-frequency characteristics of the MEMS structure must be considered [33]. In the same year, Younis et al. proposed the analytical response of a double-ended fixed beam structure under shock loads. The results show that, for microbeams fixed at both ends, there could be a nonlinear term in the response, resulting in a more complex transient process of the structure [34]. In 2009, Yagubizade and Younis et al. further studied the analytical response of the double-ended fixed-supported beam structure, based on the Euler-Bernoulli beam model, and with a Squeeze-Film damping term. The results showed that the damping term can significantly change the response of the structure [35].

In 2011, Subramanian Sundaram et al. [36] studied the response of a MEMS comb structure under shock and vibration. They proposed the concept of the valid region for the driving voltage, which could be a quantitative reference for the reliability of electrostatically driven comb structures under shock loads. The reliability prediction was further verified through experiments of 65 g in-plane and 6000 g off-plane shock loading.

In 2013, HM Ouakad [37] studied the response of a gas sensor of a beam-plate structure under shock loads. The response modeling was based on the nonlinear Euler-Bernoulli beam. In addition to the nonlinear electrostatic driving force, geometric and inertial nonlinearities were also considered. The study indicated that when the deflection of the cantilever beam exceeds about 30% of its length, the nonlinearity in the response is non-negligible. In 2019, KS Xu et al. [38] proposed a transfer function-based MEMS shock response model, which can quickly estimate the response of the structure.

### 2.2. Numerical Research

The finite element method (FEM) is the most commonly used numerical method to calculate the shock response of actual microstructures. Jordy and Younis [39] studied the shock response of a MEMS accelerometer numerically in 2007. They compared the response of the system when the Squeeze-Film damping changes, and discussed the idea of using gas damping to optimize the shock resistance of MEMS.

**Figure 4:**
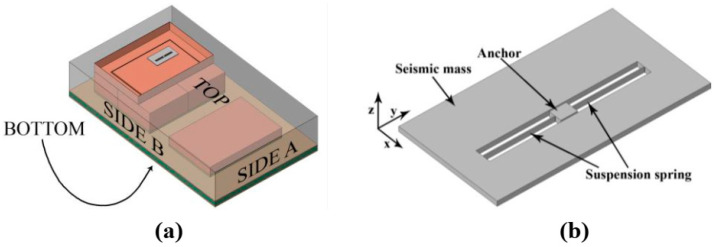
(**a**) macro-scale model of the whole package (**b**) meso-scale model of the uniaxial MEMS accelerometer [42].

In terms of numerical research of MEMS reliability under shock loads, Mariani et al. [40,41,42] have contributed in-depth studies. In 2007, Mariani et al. reported a study of a multi-scale MEMS model under a drop load, exploring the reliability of MEMS in three scales: board-level systems, MEMS structures, and polysilicon structures [40]. In the same year, Mariani et al. further discussed the multi-scale model of MEMS with modeling of the mechanical properties of polysilicon films based on tensor mechanics, and analysis of the transient acceleration and stress of the beam-mass structure under drop loads [41]. In 2011, Mariani et al. published a finite element simulation on the failure process of MEMS microbeams based on the fracture mechanics model of brittle materials, as shown in Figure 4. The research also found that, for polysilicon materials, the anisotropy of grains does not have significant influence on the MEMS structure [42]. Aldo Ghisi et al. [43] also proposed a simulation study for the drop environment of MEMS packaging in 2007. Based on Mariani’s research, the study discussed the drop resistance of the packaging structure.

In summary, the dynamic response of MEMS structures under shock loads is a complex mechanical problem. Research in 2000–2020 has made some remarkable progress, including: (1) Srikar et al. proposed the groundbreaking mechanical framework, allowing MEMS designers to determine the appropriate analytical methods for different shock scenarios. (2) Younis et al. derived analytical responses of some typical structures based on the partial differential equations in structural dynamics. Factors like the electrostatic driving force, damping, and nonlinearity were also considered, which can be good references for researchers for comparison with numerical results. (3) Mariani et al. proposed a series of numerical studies of the MEMS structure under shock loads at multiple scales. The fracture model of polysilicon from the perspective of material fracture mechanics was introduced, which provides an in-depth understanding of the impact failure process of MEMS.

However, considering the complexity of the shock response of microstructures and the relative lack of experimental observation for transient processes, existing research still has some shortcomings: (1) The accuracy of the response model has not yet been experimentally fully verified. (2) Research on the influence of different types of damping and nonlinear factors on microstructures under impact loads is relatively lacking. These issues need to be further explored in the future.

## 3. Experimental Research Progresses

The shock resistance and failure modes of different types of MEMS products can be very different. From 2000 to 2020, various experimental researches have been carried out to explore the reliability of MEMS devices under shock loads. These researches can be classified into two categories. The first category is the reliability experiment of different types of MEMS products. These experimental studies are shown in Table 4. The second category is research on improving the loading method itself.

Additionally, regarding the shock experiment of MEMS devices, industrial standards have been established to standardize the reliability testing procedures [44]. In these standards, the shock waveform is usually regarded as a half sine wave, a triangle wave or a square wave. The shock resistance levels of electronic components also have been specified.

### 3.1. Experimental Research on MEMS Products

**Figure 5:**
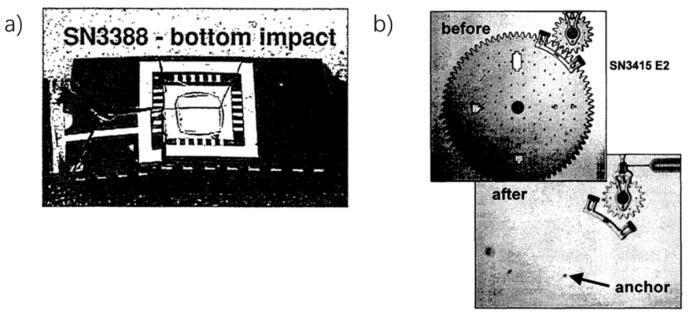
(**a**) fracture of MEMS chip under 40,000 g shock (**b**) Micro gear fracture under 20,000 g shock [46,47].

The first institutions that conducted research on MEMS under harsh environments, such as shock loads, were Sandia National Laboratory and NASA. In 2000, Tanner et al. [45,46,47], from the Sandia National Laboratory, conducted shock experiments on MEMS combs, micro gears and other typical structures. These experiments were, perhaps, the earliest to collect the failure phenomena of MEMS under impact loads (mostly fracture failure, shown in Figure 5), with systematically designed testing procedures.

In 2001, Mark S. Fan and Harry C. Shaw [48] from NASA reported a study on the reliability of MEMS accelerometers in shock environments. The study was conducted on ADXL250, which may be used in aerospace applications. These early studies used Hopkinson pressure rods as the loading equipment. The reported shock resistance of these microstructure were usually 10^3^–10^4^ g.

The DMD micromirror array is one of the most successful commercial devices in the MEMS industry. In 2002, AB Sontheimer et al. [49] conducted a systematic study on the reliability of the micromirror (especially concerning the hinge part). The failure risks (including the shock load) of the micromirror were discussed, and the life-time of the DMD was modeled.

The accelerometer is also one of the successfully commercialized MEMS products. With the micron-level size, MEMS accelerometers have the inherent advantage of resisting and measuring high-g shocks. In 2015, Narasimhan et al. [50] comprehensively overviewed existing high-g accelerometers. Many types of MEMS accelerometers can reach 10^3^–10^5^ g. However, for MEMS accelerometers with higher sensitivity and smaller measuring ranges, reliability in shock environments need to be considered. In 2012, JW Kim et al. [51] reported an experimental study on a piezoelectric inertial sensor with membrane structure, which was expected to be used in consumer electronic devices. The peak amplitude of the shock from 1.5 m could reach 2000 g. In 2015, Lall Pradeep et al. [52] conducted a shock experiment on ADXL 193 under working conditions. The study used a drop table to perform a shock load of around 3000 g. Some parameter changes were found in components, such as capacitance.

**Table 4:** Shock Experimental Research of MEMS.

Class	Products	Shock Resistance	Ref.
Class I	Accelerometer	10^4^~10^5^ g	[48,51,52]
	Microphone	>6.5 × 10^4^ g	[53,54,55]
	MEMS Inductor	>6.0 × 10^4^ g	[56]
Class II	Gyroscope	~10^3^ g	[57,58,59]
	Resonator	10^3^~10^4^ g	[60,61,62]
	Energy Harvester	~10^2^ g	[63]
	Comb Driver	~10^3^ g	[45,46,47]
Class III	Inertial Switch	10^3^~10^5^ g	[64]
	RF Switch	10^3^~10^4^ g	[65]
Class IV	Micromirror	~10^2^ g	[49]
	Gear	<2 × 10^4^ g	[46,47]

MEMS microphones are sound pressure sensors based on a membrane structure, which can be relatively fragile. Experimental studies on the shock resistance of MEMS microphones have been reported in recent years. J. Li et al. [53] conducted shock experiments on MEMS microphones in various loading directions in 2013. Optical microscopes, scanning electron microscopes (SEM), and energy dispersive X-ray spectrometers (EDS) were used to observe damage in the membrane. In 2014, J. Li et al. further combined corrosive airflow and shock experiments on MEMS microphones. It was found that the microphone’s membrane could suffer a structural embrittlement fracture and wire bond separation due to the airflow environment. However, failure phenomena, such as breakage of the microphone membrane, were not found in the shock experiments. J. Li [54] pointed out that the impact resistance of the MEMS microphone in the *Z*-axis direction (the rather fragile direction) is higher than 65,000 g. In light of the complicated stress distribution in the porous membrane structure of the microphone, C Lu et al. [55] used the Taguchi method to calculate the stress in the MEMS microphone under the impact load. An optimized design with higher sensitivity was further proposed by improving the stress concentration part.

A suspended inductor is an emerging type of MEMS device, with potential RF applications. In 2020, L Xu et al. [56] reported research on the reliability of a suspended inductor under high-g shocks. It was found that its shock resistance was above 60,000 g.

MEMS gyroscopes are generally based on resonant structures, which tend to have relatively low structural stiffness for the convenience of electrostatic driving. This feature usually makes the shock resistance of the gyroscope significantly lower than other MEMS devices, such as accelerometers. In 2012, J. Li et al. [57] reported an experimental study on a three-axis MEMS gyroscope. The package of the gyroscope used in this article failed at a horizontal shock above 8000 g, while the resonant comb structure failed by friction or fracture at a horizontal shock of around 4000 g. Ya. A. Nekrasov et al. [58] conducted environmental experiments of vibration and shock on gyroscopes with different resonant frequencies in 2015, They found that gyroscopes with higher resonant frequencies had higher reliability. In 2020, J Lian et al. [59] used the Machete Hammer to simulate the shock of gun shot on MEMS gyroscopes. The study found that the shock resistance of the gyroscope structure, under the driving signal, was lower than that in the non-working state.

MEMS resonators are widely used microstructures. Considering that they are usually driven mechanically, the stiffness of the resonator structures tends to be relatively low, and, thus, so is the shock resistance. An experimental study by L Dong et al. [60] on the shock resistance of a disc resonator structure in 2014 found its failure threshold to be around 10,000 g. Pradeep Lall et al. [61] conducted an impact experiment on the resonator in 2016 and found that the most vulnerable linkage in the structure was the conductive epoxy that connected the resonator chip to the oscillator chip. In 2017, D B. Heinz et al. [62] tested a resonant tuning fork structure manufactured by an epitaxial process. A rail-type shock equipment was customized in the study. It was shown that the resonator’s structure had a shock resistance of above 20,000 g.

Recently, MEMS energy harvesters have become a popular research field. These MEMS energy harvesters have potential applications in both industrial and wearable devices. The energy harvesters are usually designed to respond to small environmental disturbances. Therefore, the stiffness of the energy harvester tends to be very small, which makes its shock resistance poor. In 2014, T Fujita et al. [63] conducted an experimental study on the impact resistance of an energy harvester. They further optimized the reliability through the design of the buffer device. The shock resistance of the structure was approximately 400 g. Renaud, M., et al. [64] further improved the shock resistance of the energy harvester to around 2500 g in 2018.

MEMS switches will experience frequent contact, collision and friction when in a working state. The reliability of MEMS switches under shock environments is worthy of attention. In 2013, W Ying [65] reported that the shock resistance of a switch structure was above 75,000 g. In 2014, G D Pasquale et al. [66] focused on the collision and wear failure phenomenon of a MEMS RF switch. It was found that the switch was safe under collisions of less than 10,000 times.

Bio MEMS, like minute pressures sensors and microfluids devices, are thriving fields nowadays, with potential applications in future healthcare and wearable equipment. The microstructures of these MEMS products tend to be highly sensitive and could be vulnerable to shock loads [5]. However, very little attention has been paid to the reliability of these MEMS devices. Ingrid Eitzen et al. [67] overviewed the use of wearable sensor technology for detecting shock impacts in 2021. The reliability of these wearable sensors was questioned by Eitzen. It seems that further study is needed regarding this field.

### 3.2. Shock Experimental Method

**Figure 6:**
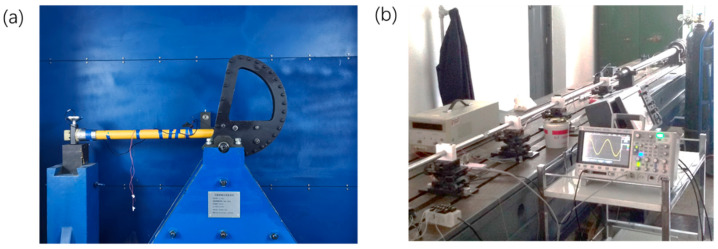
(**a**) Machete Hammer (**b**) Hopkinson Bar.

The shock experimentation of MEMS needs to provide mechanical loads of high amplitude and short duration. This poses certain challenges to the control of the shock’s waveform and the real-time data acquisition technique. Commonly used loading equipment includes a drop table, Machete Hammer, and Hopkinson Bar (shown in Figure 6). In terms of damage observation methods, optical microscopes, scanning electron microscopes (SEM) and energy dispersive X-ray spectrometers (EDS) are commonly used. The real-time observation of the mechanical and electrical response of the structure is mainly carried out through oscilloscopes and high-speed cameras. From 2000 to 2020, shock experimental methods of MEMS have made some progress worthy of attention.

Tanner et al. published a systematic study in 2000 and established early basic experimental methods for MEMS devices, including for shock and harsh environments [47]. Since then, Pradeep Lall et al. [68,69,70] have made more innovations in the shock experiment method. These progresses include a study published in 2007, in which a high-speed camera technology, based on computer image processing, was proposed, which can measure the real-time displacement of a PCB-level system [68]. A feature extraction method based on transient response spectrum analysis was proposed, which can identify failure signs in devices under shock loads [69]. In 2009, a resistance spectroscopy-based analysis method was proposed, which can monitor unobvious failure phenomena within electronic components [70].

In addition to board-level experimental methods, there are also some noteworthy developments in experimental methods. C Yang et al. [71] established a drop table system in 2010. Real shock data collected from this experiment were put into finite element analysis instead of half-sine simplification waveform. The numerical method is, therefore, better connected with the experimental results. L Zhang et al. [72] proposed a micro-hammer structure in 2016, which can provide shock loads up to 120,000 g on the micron scale. In 2017, D She et al. [73] proposed a PZT-based shock excitation device. Since this device can better control the waveform, it can calibrate the dynamic performance of the structure. In 2017, Y Cao et al. [74] used a high-speed camera device to observe the dynamic response of an inertial switch under a shock load in real time. This study captured the transient response process of a MEMS structure at the micrometer scale for the first time. In 2019, H Feng et al. [75] used a centrifuge to provide a static load of up to 23,000 g with good amplitude accuracy, which is suitable for studying the static response of MEMS inertial switches.

In summary, much progress have been made in experimental research of MEMS in shock environments during 2000 to 2020. Experimentation on the major types of MEMS products have been conducted in order to observe their shock resistance. At the same time, the shock excitation method and the real-time observation method have also made a lot of progress, providing the basic conditions for studying the reliability of MEMS in shock environments.

However, experimental research in this field also has some problems that need further study. In terms of the shock resistance of the MEMS devices: (1) The shock resistance test results in different types of devices still lack statistical evidence; (2) Some innovative MEMS structures still need shock experimental research. In terms of experimental methods: (1) There is lack of dynamic observation methods for the displacement, strain, and stress of the microstructure; (2) There is a need for excitation methods of high-amplitude, low-frequency, and high-quality half-sine pulse. These issues need to be studied in the future.

## 4. Shock Resistant Microstructures

Generally, MEMS devices tends to have better shock resistance than traditional sensors and actuators. However, it can be seen from Table 4 that for some MEMS structures with low stiffness and low characteristic frequency, such as gyroscopes, energy harvesters, switches, resonators, actuators, and others, the shock load with magnitude of 10^2^~10^4^ g could cause damage to the microstructure. Such shocks are common in industrial, aerospace or military applications. Therefore, it is necessary to optimize these microstructures for better shock resistance.

During 2000–2020, there has been a lot of research in the field of MEMS shock-resistant structure design. This progress can be classified into three categories: stoppers, latch mechanisms, and specific anti-shock structures.

### 4.1. Stoppers

**Figure 7:**
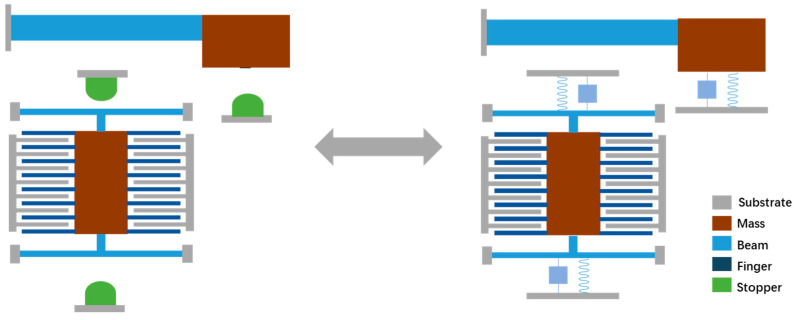
The diagram and spring-damping model of the MEMS stopper.

Stoppers are commonly used in MEMS designs to enhance structure reliability. According to the second strength theory, when the strain of brittle materials exceeds the maximum strain, structural fracture will occur [76]. By changing the boundary conditions of the structure, MEMS stoppers can prevent excessive strain in the structure, as shown in Figure 7. Therefore, this is one of the most effective designs for preventing fracture failure caused by the shock load. The research on MEMS stoppers mainly focuses on three areas.

The first area is the study of the mechanical response model of the stopper. Naumann et al. [77] reported early modeling of the collision process of MEMS stoppers in 2010. Jiang, Tao et al. [78,79,80] reported dynamic modeling on the response of MEMS stoppers under shock loads in 2012, 2014, and 2018, and proposed a parametric design method for stoppers. In 2017, Lehée, Guillaume et al. [81] modeled the collision failure phenomenon of MEMS stoppers under shock loads. They further predicted and experimentally verified the load limit that the stopper can withstand.

**Figure 8:**
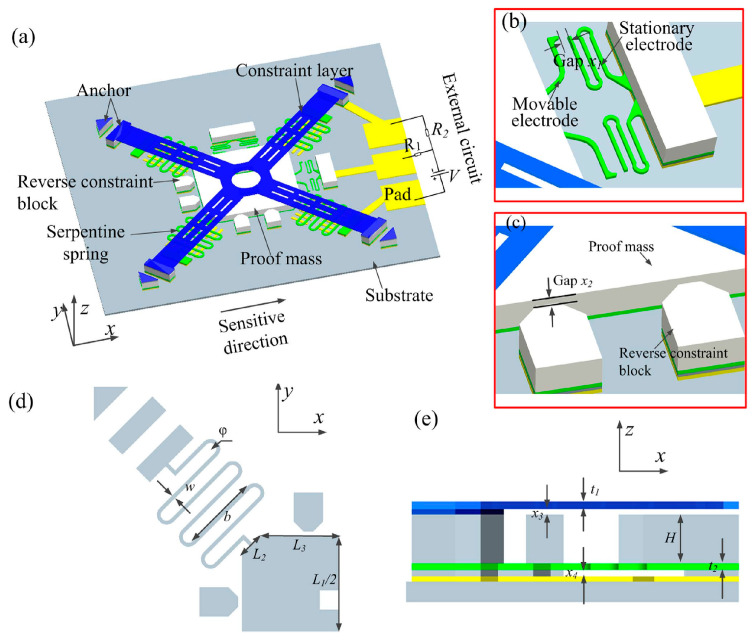
(**a**) The MEMS switch with stopper structure. (**b**,**c**) The details of the switch. (**d**) The top view of the switch’s electorde. (**e**) The side view of the structure [83].

The second area is the innovative design of the stopper. Tao, Yong-Kang et al. [82] proposed a multi-stage stopper structure for dual-mass gyroscopes in 2014, which increased the shock resistance of the structure to about 10,000 g. In 2017, Xu, Qiu et al. [83] reported a shock-resistant MEMS design with stoppers in all three-axis directions, as shown in Figure 8.

The third area is the optimization of the material of the stoppers. AK Delahunty et al. [84] proposed a MEMS stopper made of metal in 2014, which increased the shock resistance of the structure to about 6000 g. In 2016, Y Daisuke et al. [85] modeled and analyzed the shock resistance of a three-dimensional stopper structure based on a multilayer metal process. The impact resistance of this MEMS structure was about 11,000 g. In 2017, ST Chen et al. [86] explored the use of air damping as a soft limiter for shock resistance. In 2019, Lani et al. [87] proposed a 3D printing-based stopper, using the cushioning characteristics of low-rigidity materials to explore the application of additive manufacturing in MEMS manufacturing. In this study, the shock resistance of the MEMS micromirror was increased from 278 g to approximately 976 g.

### 4.2. Latch Mechanisms

The latching mechanism is a multi-stable mechanical structure. The latching mechanism buckles up the microstructure when it is subjected to a shock load, thereby achieving better shock resistance. The protection effect of the latch is one-time, since it is difficult to restore the structure to the original state after being buckled. However, the latch mechanism still has potential application in products like MEMS-based safety and arming devices.

**Figure 9:**
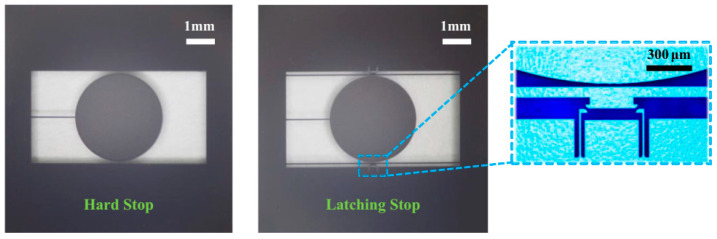
Processing and pictures of anti-shock latching structure [89].

Kaisi Xu et al. [88,89] reported a stopper based on the latch structure in 2016 and 2018, which can protect the structure from damage by self-locking under shock loads. The latching structure proposed in this study can reduce about 2/3 of the structural impact (as shown in Figure 8 and Figure 9). Lee and Yeonsu [90] proposed a latching inertial switch with a threshold of about 44 g in 2016. In 2018, H Tu et al. [91] used FORM (First Order Second Moment Method) to measure the working reliability relative to the variability of size parameters. In 2019, H Feng et al. [75] reported a latching inertial switch for arming devices. The reliability of the structure exceeded 50,000 g. Singh et al. [92] conducted an in-depth study on the latch structure in 2020. A quantitative model for the trigger threshold of the latch structure was proposed, and verified through the centrifuge experiment.

### 4.3. Specific Anti-Shock Structures

**Figure 10:**
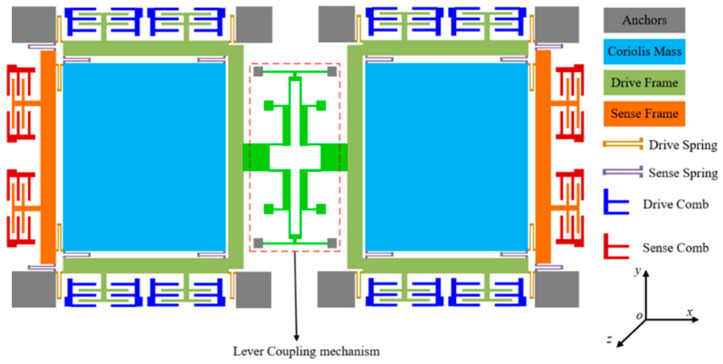
A shock-resistant dual-mass gyroscope [94].

In addition to the stopper and the latch mechanism, there have also been some innovative structural designs specifically for shock resistance in recent years. The most representative of these is the optimized design of the dual-mass coupled gyroscope. In 2014, J Zhou [93] proposed a lever coupling mechanism in a dual-mass gyroscope, which increased the shock resistance in the driving direction to more than 10,000 g. Y Gao and M Fathalilou [94,95] also proposed a mechanical structure resistant to high-g shocks based on the dual-mass model in 2017 and 2020, respectively (as shown in Figure 10). These studies showed that the dual-mass model could significantly improve the impact resistance of the driving direction without sacrificing structural sensitivity.

S Yoon [96] published a study outlining a tactical-level MEMS ring shape gyroscope in 2005, which uses a ring resonator structure to reduce the stress concentration problem of traditional resonators. B Bai et al. [97] proposed a shock-resistant MEMS hydrophone in 2017 in which the structure’s shock resistance could reach 2000 g. In 2018, F Liu et al. [98] reported an optimized design of an accelerometer in a high-g environment based on the Timoshenko beam theory. The shock resistance of the structure reached 200,000 g without sacrificing high sensitivity.

In summary, the progress of shock resistant microstructures from 2000 to 2020 has occurred mainly in three areas, namely, stopper, latch structure and specific anti-shock structure. The stoppers are, perhaps, the most effective and simple anti-impact structure, and have been widely used in actual commercial products.

However, for MEMS structures with low stiffness and low characteristic frequency, poor shock resistance still limits the application of products, such as gyroscopes and resonators, in high-g shock environments. Some of the existing optimized design schemes are also difficult to put into practice because the processing is too complicated.

## 5. Reliability Quantification Model

For applications like aerospace and defense, the reliability of the electronics component usually needs quantitative measurement or prediction based on probabilistic modeling. The reliability quantification of MEMS under shock loads involves mechanics of materials, reliability engineering, and statistics, among others. To date, we have not yet found any study that statistically predicts the reliability of MEMS devices under shock loads or provides a quantification model.

However, it is still necessary to review a neighboring field, which is the structural reliability of MEMS under static mechanical loads, so as to provide a reference for understanding the structural failure process of MEMS under shock loads. The research in this field can be classified into two areas, namely, the strength model of brittle materials and the reliability quantification model of microstructures.

### 5.1. Strength Model of Brittle Materials

For the numerical research of the reliability of MEMS under shock loads, parameters like the Young’s modulus, Poisson’s ratio, and tensile strength of materials are very important. Many researches have measured these parameters [99,100,101,102,103,104,105,106]. It is worth noting that these studies have shown that the parameters, like the tensile strength of MEMS materials, can fluctuate in a rather wide range.

**Figure 11:**
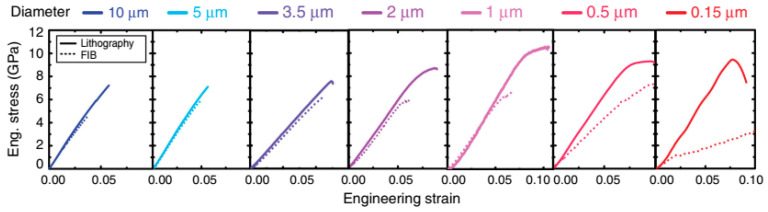
Single crystal silicon sample with different size and process exhibited different strength [107].

The tensile strength of the material is probably the most important parameter for determining the shock resistance of the microstructure. However, studies have shown that the tensile strength of MEMS materials are significantly affected by factors, such as loading method, measurement method, processing technology, and structure size [100]. Taking polysilicon material as an example, the measured tensile strength can fluctuate between 2–4.5 GPa, depending on the size, shape, and process of the device [103]. The tensile strength of single-crystalline silicon material fluctuates even more. According to previous studies, the tensile strength could be between hundreds of MPa and 4 GPa (shown in Figure 11) [107].

The variation of the tensile strength of MEMS materials makes it difficult to predict the failure threshold or quantify reliability through numerical analysis. Therefore, some studies have proposed probabilistic models for MEMS structural materials, which regard the tensile strength of the material as a random variable. By this mean, the numerical study of structural reliability of MEMS can have statistical significance.

A.M. Fitzgerald et al. [103] measured the fracture strength of single crystal silicon materials in 2002. The experiment showed that the $K_{Ic}$ of single crystal silicon samples is 1.15 ± 0.08 *Mpam*^0.5^ [108]. Brad L. Boyce et al. measured the strength distribution of polysilicon materials in 2007 and found that the strength is significantly affected by temperature. Mohamed E. Saleh et al. [109] conducted tensile experiments on the tensile strength of polysilicon in 2014. The tensile strength of the sample was between 2 GPa and 4.5 GPa, and was in good agreement with the three-parameter Weibull distribution. Frank W. DelRio et al. [110] reported a comprehensive review of the fracture strength of nano-scale single-crystalline and polycrystalline silicon materials from the perspective of crystal structures in 2015. In 2018, Robert F. Cook et al. [111,112] published an in-depth mathematical modeling of the strength of brittle materials based on the statistical characteristics of material microscopic defects. The results of the model are in good agreement with the results of the tensile experiment.

**Figure 12:**
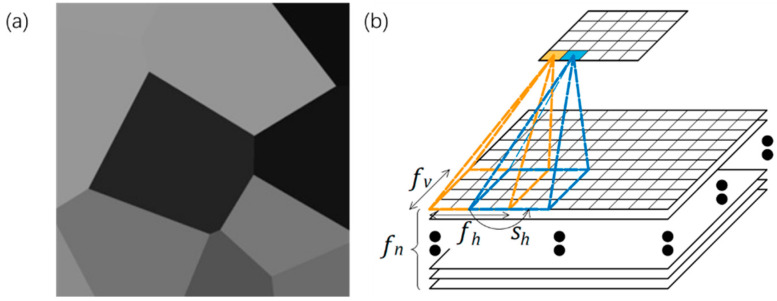
(**a**) morphologies of statistical volume elements (**b**) schematic of the receptive field of a convolutional layer [115].

In 2016, Vayrette, Renaud et al. [113] conducted fracture experiments on polysilicon films with a thickness of 40 nm and 240 nm. They found that the micro-grooves between the grains are the critical reason leading to structural fracture. Mariani et al. [114,115,116] produced a series of studies. In 2011, Mariani et al. performed multi-scale modeling of MEMS structures. Modeling of polysilicon materials at the crystal scale showed that the micro-cracks between grains are the main cause of structural fracture [114]. In 2019 and 2020, José Pablo Quesada Molina et al. used deep learning to characterize the mechanical properties of polysilicon materials based on the data set (which was generated numerically, as shown in Figure 12). The deep learning model could predict the relationship between the grain characteristics and the mechanical properties of polysilicon [115,116].

### 5.2. Reliability Quantification Model

In addition to the probabilistic model of the tensile strength of MEMS materials, the quantification model of the structural reliability of MEMS could have more direct influence on industrial applications. Some early studies analyzed the fracture load threshold of the structure based on the basic partial differential equation of the microstructure [117]. However, more accurate reliability quantitative models were not been reported until the last ten years. Research in this field can be roughly classified into three areas: quantification models for general MEMS structures, models based on Weibull distribution, and weakest-link models. The key calculation formulas of each model are shown in Table 5.

• Reliability Quantification for General Structures

Reliability Quantification for General Structures refers to studies modeling structural reliability, regardless of the actual design of microstructure. Chen et al. proposed the concept of the reliability quantification model in 2003, referring to it as the “equivalent strength model.” The main idea behind this model is to derive the reliability of the entire structure based on the strength distribution of the micro-element within the MEMS structure [118]. Alissa M. Fitzgerald proposed the first reliability quantification model for a general MEMS structure in 2009. Through the integration of failure probability on each surface of the MEMS structure, the overall reliability of the structure is obtained [119,120]. In 2011, Fitzgerald’s model was further explored by proposing the concept of structural failure probability density (FPI). According to the stress distribution on the surface of the structure, the FPI quantified the failure probability of different parts of the microstructure [121]. Based on Fitzgerald’s model, K Nagayoshi obtained similar results through stretching and bending experiments on single crystal silicon samples in 2010 [122]. Gilad Sharon et al. further carried out statistically significant experimental research through sample arrays of monocrystalline silicon in 2013 [123].

• Weibull Distribution Model

**Figure 13:**
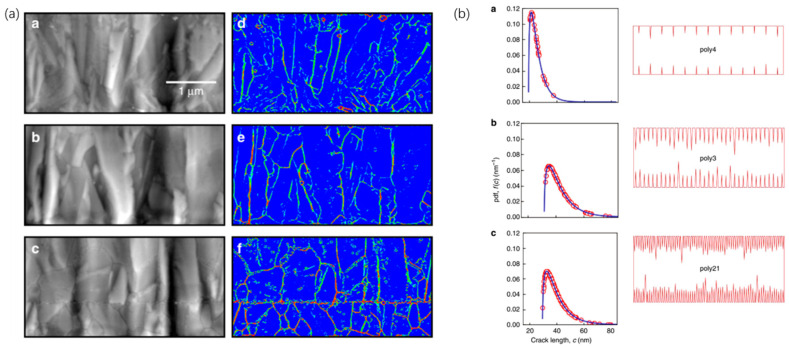
(**a**) Defects on the sidewall of the sample observed by AFM (**b**) The structural strength predicted based on the statistical distribution of the surface defect [126].

Weibull distribution is a commonly used statistical distribution for the tensile strength of ceramics and other brittle materials. Sandia National Laboratory has done a wealth of work on the structural reliability model of polysilicon materials based on the three-parameter Weibull distribution [110,111,112]. Furthermore, the research team of Sandia National Laboratory applied the Weibull distribution to predict the reliability of basic MEMS microstructures in recent years. In 2010 and 2011, E. D. Reedy et al. [124,125] used the three-parameter Weibull distribution to predict the distribution of the fracture threshold in the polycrystalline silicon tensile beam structure (with approximately 1500 samples). Optical microscopes, Confocal Raman Microscopes (CRM), and Atomic Force Microscopes (AFM) were used to observe the structure during stretching and after fracture failure. The results show that, for the samples made by the Summit V process, the characteristic depth of the surface defects is 25–61 nm, with an average of 35 nm, indicating that surface defects lead to fluctuations in the fracture strength of the polysilicon. In 2019, Robert F. Cook et al. [126] further proposed the distribution of the fracture threshold based on the characteristic scale of surface defects on the tensile fracture of the sample. Through the atomic force microscope (AFM) observation of the defects, it was found that the model successfully reflected the relationship between the reliability of the microstructure and the material-level characteristics, as shown in Figure 13.

At the same time, Oscar Borrero-Lopez et al. [127] used the two-parameter Weibull distribution to conduct an experimental study on the fracture strength of the membrane structure. They found that the pressure threshold of the membrane followed the Weibull distribution as well.

• The Weakest-link Model

The weakest-link model is a commonly used model from system reliability theory. It is suitable for describing the reliability of a series system. Jia-Liang Le, Zhifeng Xu et al. [128,129,130,131] proposed several reliability models for polysilicon MEMS structures. Jia-Liang Le et al. first proposed a definite weakest-link model in 2015. The strength prediction based on this model was in good agreement with the experimental results of Sandia National Laboratory [128]. In 2017 and 2019, Zhifeng Xu et al. proposed a renewed model based on the First Passage Analysis. The study found that this model is an extension of the Weibull distribution [129,130,131]. The weakest-link model constructs a framework for the fracture of MEMS based on system reliability theory. It also establishes the connection between the macro-level data of the tensile experiment and the micro-level characteristic obtained from the microscopic observation.

**Table 5:** Reliability quantification model of microstructure.

Reliability Model	Key Equations	Ref.
Reliability Quantification for General Structures	$P_{s}\left(A\right)=\exp\left[-\int_{A}\Psi dA\right]$	[119,120,121]
Weibull Distribution Model	$P=1-\exp(-{\left(\frac{\sigma -\sigma_{u}}{\sigma_{\theta }}\right)}^{m})$	[124,125,126,127]
The Weakest-link Model	$P_{f}\left(\sigma_{N}\right)=1-{\left[1-\int_{0}^{\infty }F_{f_{t}}\left(x\sigma_{N}\right)f_{s}\left(x\right)dx\right]}^{2n}$	[128]
	$P[\eta_{0}(x)\leq \lambda ,\forall x\in L]=F_{\eta_{0}}(\lambda )\exp[-\frac{\mu_{\lambda }}{F_{\eta 0}(\lambda )}L]$ $where:f_{\eta_{0}}(\eta )=\int_{-\infty }^{+\infty }\sigma_{N}^{-1}f_{Z_{0}}(z/\sigma_{N})f_{f_{t0}}(z-\eta )dz$	[129]
	$P_{f}\left(\sigma_{N},L\right)=1-{\left[{\bar{R}}_{e}\left(\sigma_{N}\right)\right]}^{2\bar{k}}$	[130]

In summary, research on the tensile strength of MEMS materials and structural reliability has made great progress from 2000 to 2020. The fracture strength distribution of MEMS materials, as well as probabilistic reliability of typical structures under static loading, are studied. These progresses provide a reference for the fracture threshold of MEMS under shock environments.

However, current research is still focused on simple structures and static loading. Research about the practical structure or the reliability prediction under dynamic load is still needed.

## 6. Electronics-System-Level Reliability

Most studies on MEMS devices under shock loads have simplified the shock to a certain extent. For example, some studies have regarded the shock loads as half-sine, triangular, or square wave loads [30,36]. Additionally, the shock test standards also use half-sine waves as a standard experimental procedure for the shock resistance of MEMS devices [44]. The advantage of simplification of the shock loads is that it makes the research more convenient and standardized.

However, this simplification of the shock waveform also brings problems. That is, this simplification does not always reflect the actual transmitting process of high-g shocks. In actuality, the acceleration pulse that acts on the microstructure is influenced by many factors, such as the mounting conditions, package type, and others.

The real working environment of microstructures is a system composed of substrates, leads, packages, and PCBs, among others. The shock load could change significantly when transmitted through these structures. Insufficient understanding of the transfer process of the shock in real working conditions may cause two types of problems. First, in relatively safe working conditions (in which the shock load is buffered by the system where the MEMS is mounted), the reliability of the MEMS device may be over-designed, thereby sacrificing part of the device performance. Secondly, under harsh working conditions (in which the shock load is amplified by the system where the MEMS is mounted), MEMS devices considered safe under the simplified assumption may be actually damaged.

Therefore, it is necessary to study the system-level transfer process of MEMS devices under high-g shock loads. Research in this field mainly focuses on three aspects, namely: system-level experimental research of electronic equipment in shock environments, system-level modeling and optimization design for shock loads, and research on key factors of shock load transmission.

### 6.1. System-Level Experimental Research

**Figure 14:**
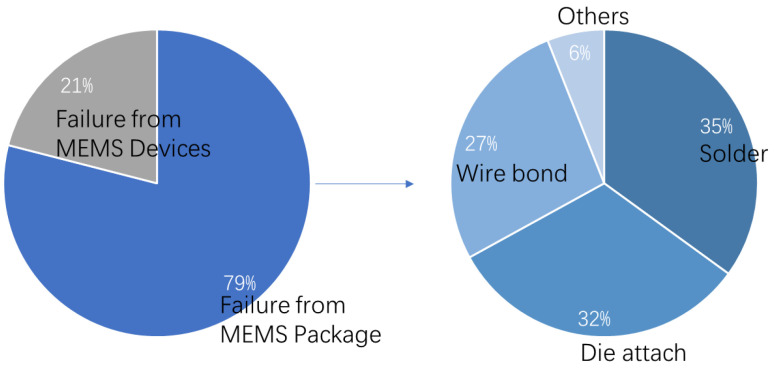
Percentage of failure sites for all 20,000 g tests [136].

System-level experimental research of shock is the usually the first step to look into the reliability of electronics equipment. Pradeep Lall [132,133,134,135] has made a lot of progress in this field. In the past 20 years, his research team has produced a rich series of studies, such as the visual strain measurement system of board-level electronic equipment, failure signal identification methods, system health monitoring, and shock resistance of electronics, among others. A lot of experimental data has been reported about the response and failure phenomena of electronic systems under shock loads. The University of Maryland also has achieved noticeable progress in this field. In 2016, J. Meng et al. [136,137] conducted a comprehensive experimental study on the reliability of MEMS in board-level systems under shock loads. The study found that about 79% of the failures occurred in the MEMS package, and 21% of the failures occurred in the MEMS silicon part, as shown in Figure 14.

In addition, TC Chai et al. [138] proposed a board-level drop environment reliability analysis model for IC packaging in 2008, which analyzed the strength of the solder joint at different positions on the PCB. F Alsaleem et al. [139] reported a study about the influence of PCB structure on the response of the mounted MEMS under shock loads in 2010. J Cui et al. [140] studied the reliability of the board-level system under high-g shocks in 2011. The research indicated that solder joints are the key fragility in the system in shock environments. Suggestions to improve the shock resistance of MEMS were also proposed.

### 6.2. System-Level Modeling and Optimization

Researches on system-level modeling and optimization focus on describing and improving the transfer characteristics of electronic systems based on lumped mechanical system models. C. Zhou et al. [141] proposed a shock-resistant model for mobile electronic devices in 2009. In 2011, J. Wang et al. [142] analyzed the shock load transfer model of electronic equipment in the three-axis direction using the shock response spectrum (SRS). In 2018, Baranyai et al. [143] proposed a mechanical model and anti-drop design of electronic systems. In 2020, Y. Shi et al. [144] published a system-level modeling of MEMS accelerometers under high-g shocks. This paper indicated that the potting treatment could significantly increase the shock resistance of the system by increasing its natural frequency and changing its stress distribution.

### 6.3. Key Factors of Shock Load Transmission

Chip packages, solder joints, and potted structures are the major factors that influence the transmission of the shock load. A great number of studies and monographs have been published on the reliability of chip packages and solder joints [145,146,147]. Along with the development of packaging technology, more studies on the reliability of packages and solder joints are being reported. However, among these published studies, only a few focus on the reliability of packages under shock loads [148]. In terms of the reliability of the potted structures, Pradeep Lall et al. studied the mechanical properties and impact resistance of the PCB-epoxy interface in 2018 and 2019 [134,149]. S. A. Meguid et al. [150] conducted a study on the shock resistance of double-sided potted PCB structures in 2014. The U.S. Army Armament Research, Development and Engineering Center (ARDEC) has also carried out a series of finite element simulation studies on the shock resistance of potted structures in recent years. A system-level polymer shock cushioning structure was also developed [151,152,153,154].

In summary, current studies on electronics-system-level reliability in shock environments are mainly experimental. Some optimizations have been proposed to protect the MEMS and other chips in the system.

However, electronics systems that can withstand a shock load of above 10,000 g could only perform relatively simple functions, like some fuses for ammunitions. It is still difficult to for electronics systems to accomplish tasks, such as multi-dimensional data sensing and processing. This reflects that further research is needed on shock transmission, weak links and anti-shock methods of the system where the MEMS device is located.

## 7. Coupling of Shock with Other Factors

Shock is often accompanied by other harsh environments in real work conditions. Some common coupling factors include: heat, electromagnetic, salt spray, corrosion, and radiation, among others. These coupling effects make it more difficult to describe the reliability of MEMS. Sometimes it is difficult to locate the primary reason of failure among these environmental challenges.

Research on the coupling effects of other harsh environments will help to understand the reliability of MEMS devices in real working conditions. Previous research in this field has mainly focused on three areas, namely, electrical-shock coupling, thermal-shock coupling, and multi-factor reliability models.

### 7.1. Electrical-Shock Coupling

The shock reliability of MEMS with electric driving force is the most common coupling problem. The MEMS structure driven by electrostatic force usually has low stiffness, and, thereby, is prone to large displacement or even fracture in shock environments. At the same time, electrostatically driven MEMS devices could suffer pull-in effects, which is more likely to happen in a shock environment.

Younis et al. produced rich research in this field. In 2006 and 2007, Younis et al. [155,156] published the modeling and analysis on the response of electrostatically driven microstructures under shock loads, including clamped–clamped microbeams and capacitive switches. These studies found that electrostatic force significantly reduces the reliability of the structure under shock loads. Specifically, the transient response can cause instability and even short failure in the system. Damping was also found to effect the reliability significantly. H M Ouakad and J D Alqasimi et al. have also reported a series of studies focused on electrostatically driven cantilever beams (2010) [157] and electrostatically driven bi-stable switches (2017, 2018) [158,159]. In 2020, K. Larkin et al. [160] published analysis and numerical modeling around microcracks in a cantilever beam structure driven by static electricity. The analytical model and the numerical model agreed well in the paper. In 2021, Kevin, L et al. [161] proposed a multi-fidelity modeling on the electrostatically-actuated cracked micro-beams.

### 7.2. Thermal-Shock Coupling

The coupling effect of temperature and shock load is also an important factor affecting the reliability of the system. Evidence has shown that the mechanical properties of the MEMS structure will change at different temperatures [105]. Research on the thermal-shock coupling is very necessary, considering that temperature cycles and shock environments are very common in aerospace and industrial applications.

In 2013, AS Haynes [162] reported a study on the reliability of the potted fuse under coupling effect of temperature and shock. The research found significant changes in the mechanical character of potting material at high temperatures (71 °C) and low temperatures (<51 °C), which affect the reliability of the system. Pradeep Lall et al. studied the reliability of MEMS devices under multiple factors, including heat and shock, in 2014. The paper reported that the temperature cycling and high humidity environments would reduce the structural reliability of MEMS packages [163].

### 7.3. Multi-Factor Reliability Models

When multiple factors of harsh environments act simultaneously, it is sometimes difficult to determine which factor presents the most significant risk of failure. Failure-Envelope Approach to Modeling (FEAM) is perhaps the most commonly used method to locate the critical factor of failure [132]. In 2008, Yuanbo Li et al. [164] provided a brief overview of the reliability research methodologies of MEMS. Qian F et al. [165] investigated the reliability analysis for multiple dependent failure processes in 2010. In 2014, Zheng Zhang et al. [166] used the ANOVA (analysis of variance) method to quantify the uncertainty in MEMS devices, which could be applied in future research of multi-factor reliability models. In facing this challenge, some studies on multi-factor reliability models for MEMS were proposed in recent years. Yaqiu Li et al. [167] discussed the relevance of competing failure mechanisms in 2017.

In summary, some early studies were conducted on the coupling effects of shock loads and other harsh environments. However, due to the complexity of the coupling effect, there is currently no effective model to evaluate the reliability of MEMS under multiple harsh environments. This requires further research and exploration in the future.

## 8. Conclusions

This article reviews the reliability research of MEMS devices in shock environments during the period of 2000 to 2020. Theoretical and experimental research frameworks have been established through studies in these years. Generally speaking, MEMS devices are more shock-resistant than traditional sensors and actuators, which could endure the shock loads of the amplitude of 10^2^ to 10^4^ g. However, products like MEMS gyroscopes, micro-mirrors and energy harvesters are relatively fragile in the shock environment due to their lower stiffness. Additionally, many protective structures, like stoppers, have been developed to enhance the shock resistance of MEMS. Some system-level protective approaches have been also studied.

Despite this progress, the reliability issue of MEMS in shock environments is still a very complex problem that need further research. The dynamic response of the microstructures is worthy of attention in order to understand the transient process more accurately. Reliability problems in emerging devices, like bio-MEMS, microfluid MEMS and wearable MEMS, need to be further investigated. Additionally, reliability modelling and management of multiple coupling factors, including shock, are perhaps a more practical issue that need to be dealt with.

## Figures and Tables

**Table 1:** Shock loads in different work scenarios [1].

Scenarios	Shock Load
Ships	120~150 g, 25 Hz
Vehicles	0.1~1 g, 5~50 Hz
Industry	0.1 g, 5~100 Hz
Earthquake	0.1~0.5 g, 2~90 Hz
Drop	10 g, 50~200 Hz
Plane Crash	15~30 g, ~100 Hz
Gun Shot	10^3^~10^4^ g, 10^2^~10^3^ Hz
Hard Target Penetration	10^4^~10^5^ g, 10^3^~10^4^ Hz

**Table 2:** Classification of MEMS in reliability research [11].

Class	Features	Products
Class I	No movable/active structure	Accelerometer, pressure sensor, micro injection pin
Class II	Active structure, no contacts or frictions	Gyroscope, resonator, filter, comb driver
Class III	Active structure with contacts but no frictions	RF switch, micro valve
Class IV	Active structure with contacts and frictions	Optical switch, micromirror

**Table 3:** Typical failure of MEMS [12].

Class	Features	Failure
Class I	Accelerometer	wear, fatigue, fracture, electrical failure
	Pressure sensor	fracture, fatigue, shock, vibration
Class II	Gyroscope	shock, vibration, electrical failure
Class III	Heat Actuator	wear, shock, vibration
	Micro Valve	wear, fracture, fatigue, shock, vibration
	RF Switch	wear, fracture, fatigue, shock, vibration, electrical failure
Class IV	Electrostatic actuator	wear, fracture, fatigue, shock, vibration, friction
	Optical switch	wear, fracture, fatigue, shock, vibration
	Micromirror	wear, fracture, fatigue, shock, vibration, optical performance degradation
	Micro gears	wear, fracture, fatigue, shock, vibration, friction
	Micro turbines	wear, fracture, shock, vibration, friction

## Data Availability

Not applicable.
